# Developing an instrument for assessing fidelity of motivational care planning: The Aboriginal and Islander Mental health initiative adherence scale

**DOI:** 10.1186/1752-4458-8-36

**Published:** 2014-08-28

**Authors:** Phuong-Tu Prowse, Tricia Nagel

**Affiliations:** Student/ Clinical Psychologist Menzies School of Health Research Monash University: School of Psychiatry and Psychological Medicine, Wellington Rd, Clayton, VIC 3800 Australia; Menzies School of Health Research and Charles Darwin University, PO Box 40196, Casuarina, Australia

**Keywords:** Motivational care planning, Adherence scale, Treatment fidelity, Australian aboriginal and torres strait islanders, Instrument development

## Abstract

**Purpose:**

The aim of this study was to design and trial an Adherence Scale to measure fidelity of Motivational Care Planning (MCP) within a clinical trial. This culturally adapted therapy MCP uses a client centered holistic approach that emphasises family and culture to motivate healthy life style changes.

**Methods:**

The Motivational Care Planning-Adherence Scale (MCP-AS) was developed through consultation with Aboriginal and Islander Mental Health Initiative (AIMhi) Indigenous and non-Indigenous trainers, and review of MCP training resources. The resultant ten-item scale incorporates a 9-Point Likert Scale with a supporting protocol manual and uses objective, behaviourally anchored criteria for each scale point. A fidelity assessor piloted the tool through analysis of four audio-recordings of MCP (conducted by Indigenous researchers within a study in remote communities in Northern Australia). File audits of the remote therapy sessions were utilised as an additional source of information. A Gold Standard Motivational Care Planning training video was also assessed using the MCP-AS.

**Results:**

The Motivational Care Planning-Adherence Scale contains items measuring both process and content of therapy sessions. This scale was used successfully to assess therapy through observation of audio or video-recorded sessions and review of clinical notes. Treatment fidelity measured by the MCP-AS within the pilot study indicated high fidelity ratings. Ratings were high across the three domains of rapport, motivation, and self-management with especially high ratings for positive feedback and engagement, review of stressors and goal setting.

**Conclusions:**

The Motivational Care Planning-Adherence Scale has the potential to provide a measure of quality of delivery of Motivation Care Planning. The pilot findings suggest that despite challenges within the remote Indigenous community setting, Indigenous therapists delivered therapy that was of high fidelity. While developed as a research tool, the scale has the potential to support fidelity of delivery of Motivation Care Planning in clinical, supervision and training settings. Larger studies are needed to establish inter-rater reliability and internal and external validity.

## Background

The *1989 National Aboriginal Health Strategy* helped garner support from the wider community to improve the overall health and well-being of Aboriginal and Torres Strait Islander people (hereinafter referred to as Indigenous Australians). Specifically, the strategy identified the need for enhanced health research, a broadening of funding programs, and promotion of understanding within the workforce [1]. There is consensus amongst many academics and health professionals that Indigenous Australians continue to experience markedly poorer health outcomes than the wider community [2–4]. Dawson [5] and Clayer and Divakaran-Brown [6] have highlighted the on-going barriers that prevent Indigenous Australians from successfully engaging and accessing health services. For example: unreliable transport, lower socio-economic standing, language barriers, and lack of engagement within the health system. In addition, Indigenous Australians have higher rates of self-discharge from hospital against medical advice [6, 7] and tend to either not engage or only briefly engage with mental health professionals [8].

In the Northern Territory, researchers have sought to address these barriers to engagement of Indigenous Australians within the health system [9–11]. Two key barriers to achieving robust health outcomes are identification of effective and culturally appropriate means for transferring research into practice, [10] and the limited number of measures available to assess the quality of service delivery [12].

The *Northern Territory Aboriginal and Islander Mental health initiative* (AIMhi) aims to promote access of Indigenous Australians to mental health services through development of culturally adapted resources and training tools [13]. These tools include the development of a brief therapy entitled ‘Motivational Care Planning’ (MCP). The therapy incorporates motivational interviewing and problem solving principles. It adapts to cross cultural and socially disadvantaged settings by having a central focus on family rather than individual, and by the use of plain English pictorial tools, which guide the strengths based therapeutic approach [14].

A number of joint studies have suggested there are benefits to the adoption of this model for Indigenous mental health clients [9, 11, 14, 15]. The MCP included a series of training sessions delivered to mental health professionals and community leaders using adapted assessment and psycho education tools. Specifically, the program aimed to present strategies to strengthen cross-cultural partnerships, enhance engagement between services, clients and their local communities, and offer an effective, practical, and simple brief therapy for those with mental health concerns. The recognised strengths of the MCP led to its inclusion in a number of best practice guidelines and resources [16–22]. The MCP approach continues to be embedded in local and national contexts including conversion to an iPad application, however there is limited evidence that this program implementation is consistent with the originally developed protocol.

The past decade has seen a marked increase in psychotherapy research evaluating fidelity and adherence instruments that assess treatment efficacy. One such instrument is the treatment manual. In the 1990s the effectiveness, appropriateness and capacity to incorporate treatment manuals into similar training programs were explored [23]. It is well documented that the inclusion of a treatment manual in isolation does not necessarily guarantee the effective inculcation of a treatment method [24, 25]. Several researchers continue to explore possible methods for most effectively and efficiently transferring research into a clinical setting [26–28] to maximise the realisation of positive program outcomes [25, 28–30]. The National Institute of Health - Behavior Change Consortium (NIH-BCC) moved beyond treatment manuals to consideration of therapist delivery in conjunction with client knowledge and skill. The NIH-BCC based fidelity evaluation on a conceptual model [31], which assessed therapist delivery of treatment; the ability for the client to understand how to use the learned skills; and how well the client can apply skills.

Research into the use of fidelity tools (such as adherence scales and competence scales) is gaining increased importance in the area of clinical trials [32–34]. Including fidelity measures provides a method to document deviations from an intended model and variations within a model [35]. For meta-analyses, having inbuilt fidelity measures can assist in producing meaningful comparisons of delivered treatments [34, 36, 37]. Applying fidelity criteria in randomised clinical trials can ensure the experimental treatment is absent in the control condition [38]. These measurement methods include observational instruments specific for their therapy technique (eg. specific verbal behaviors in sessions) [39].

Researchers [40, 41] have described three steps to establishing effective fidelity criteria: identification of critical components of a given model (often based on expert consensus or the existence of a proven model through randomised control trials) [32, 42, 43]; use of a multi-method or multi-informant approach to identify key components [44–46]; and examination of the indicators in terms of reliability and internal and external validity [33].

This paper reports on the development and implementation of the Motivational Care Planning-Adherence Scale (MCP-AS). This fidelity measure supported examination of the consistency and quality of the delivery of MCP within a clinical trial based in a remote Indigenous community setting.

## Method

### Objectives

This study developed and tested an adherence scale for application within a clinical trial to measure fidelity of the experimental treatment (Motivational Care Planning) and to document deviations from the intended treatment model. The clinical trial aimed to test the effectiveness of Motivational Care Planning for clients at risk of depression or depression comorbid with substance misuse [47]. The adherence scale was one of a suite of activities seeking to promote researcher/therapist adherence during the course of the trial. It was used as a means for promoting feedback and discussion within the research team focused on issues of fidelity. It is acknowledged that the tool itself had not been validated prior to commencement of the study, but it was deemed a helpful way of monitoring the delivery of the intervention by the researchers in the absence of an alternative. Additional activities aimed at promoting fidelity included team review of the hard copy reports of the intervention and team discussions about the approach to treatment.

### Initial clinical trial design

The clinical trial was conducted in two remote Northern Territory communities. It initially used a wait list design over two periods of six months, and followed participants for a further six months. The research team aimed to deliver ‘early treatment’ at baseline while local Indigenous workers would deliver ‘late treatment’ at six months under researcher supervision. Treatment involved two 30 minute ‘Motivational Care Planning’ sessions with client and carer. The project received approval from the Human Research Ethics Committee of the Northern Territory Department of Health and Families and the Menzies School of Health Research.

### Research team

The team included a non-Indigenous psychiatrist, project manager and three Aboriginal and Torres Strait Islander research officers who conducted consultation, recruitment, assessment and delivered therapy in the remote communities. The Aboriginal and Torres Strait Islander research officers (two female and one male) had between one to five year experiences in MCP delivery. One was an experienced mental health worker, whilst the remaining two had no formal mental health training. The team was chosen to allow the treatment to be delivered by a primary therapist of the same gender as the client. This was recommended through consultation. For male clients the male research officer led the treatment, and for female clients one of the female research officers led treatment. An independent program assessor was utilised to develop a fidelity measure to monitor the treatment delivery through the review of 10% of audio recordings of treatment sessions.

### Motivational care planning intervention

Individual treatment sessions of approximately thirty minutes were conducted with the client (supported by carers) by two research officers, one of whom delivered the therapy and the other took written notes. Therapy was preceded by completion of outcome measures. Therapy sessions included identification of strengths, stressors, family and support networks, observation of a motivational video narrated by a Larrakeah traditional owner, provision of information, and establishment of goals to effect meaningful change. The story of this traditional owner engaging in key steps of the therapy was shown on an iPad, which was also used to audio record the treatment session following completion of baseline outcome measures.

### Participants

The clinical trial recruited participants from within targeted communities and the local health centres. Inclusion criteria required participants to be over the age of 12 years and screened at risk of depression and/or substance misuse. Persons identified with other mental health conditions, deemed not at risk of depression, or unable to give informed consent were excluded.

The PHQ 2+, a brief version of the PHQ-9, was used as a screening tool [48]. If screened participants measured at risk of depression they were included in the project. The PHQ-9 was the chosen primary outcome measure. It is a brief measure of severity of depression that has shown diagnostic, criterion and construct validity with Indigenous youth and adults [49]. The study aimed to recruit 154 participants.

### Revised study design

Recruitment challenges in both communities led to revision of the trial design to a detailed descriptive study. Only 39 participants were successfully recruited for screening. Of the 39 people screened only 16 met the criteria for inclusion. Of these 16 participants, nine were randomised to the treatment condition. Four of these treatment sessions were recorded for the purposes of measuring treatment fidelity (two males and two females).

### Adherence scale design

### Identifying and specifying fidelity criteria

The following three steps helped shape, identify and specify the fidelity criteria:

1. Review of established specific MCP model protocols and treatment processes. These had been tested within a clinical trial setting with positive treatment outcomes and subsequently delivered through 60 training workshops;
2. Analysis of feedback from the founders of MCP and review of existing literature including MCP publications;
3. Consultation with MCP trainers and incorporation of their views of the essential elements of MCP therapy into the MCP-AS.

The fidelity criteria of the MCP-Adherence Scale [50] sought to assess therapist delivery of treatment through attention to both structure and process. A *Structure* provides the framework for service delivery, and *process* determines the manner in which services are delivered [51]. The fidelity criteria for the MCP-AS include time taken to deliver individual components and demonstrated behaviours as described in the program publications.

### Development of motivational care planning-adherence scale

The MCP-AS is a semi-structured observational measurement tool to measure therapist fidelity in delivery of MCP. The scale items represent the core therapeutic goals of the treatment model and each item is allocated a score. Items are scored on a 9-point Likert –Type Scale with the following anchors: "0"= *not observed*, "1"= *slight evidence* "5"= *minimal acceptable evidence* and "9"=*fully implemented*.

The adherence scale rated each desired element across three domains; execution, frequency of inclusion and competency. The protocol provides a detailed description to support adherence and competence assessments for the individual therapeutic components [50].

### Observational coding procedures

#### Fidelity assessor

One experienced Clinical Psychologist (first author) completing her PhD studies (here in after referred to as the Assessor) was employed to design the adherence scale and code the audio recordings of the MCP sessions.

#### Treatment fidelity ratings

The Assessor listened to each audio-recorded session in its entirety. Session duration ranged from 20 to 35 minutes. The next step involved hearing the audio recording again using pause, rewind and play to confirm a rating of the session. This second step was repeated twice. All three ratings were recorded prior to a mean consensus being obtained. The Assessor documented descriptive commentary of each individual recording to assist in completion of global competence items. Global competence items were scored at the completion of the recordings.

#### File audit method

A de-identified research file of individual participants was maintained to record the details of the baseline outcome measures and intervention delivery. The research files were audited using the MCP-AS and reviewed three times by the Assessor to promote consistency and completeness. Four items (1, 2, 3, and 7) on the MCP-AS could not be completed through file audit as these items required direct observation of therapist behaviour. The quantitative data was analysed using the SPSS Statistical Programming [52].

## Results

Audio recordings of the four therapy sessions with Indigenous clients (two males and two females) were analysed. The ten item MCP-AS and accompanying protocol was completed with fidelity criteria across three domains: rapport (items 1 – 4), motivation (items 5–7) and self-management (items 8–10).

Overall, researchers demonstrated high levels of treatment fidelity for the majority of MCP-AS items, especially when scores were adapted following review of file notes.

### Gold standard comparison

The Gold Standard video demonstration of MCP achieved maximum scores on each item when assessed using the MCP-AS.

### File audits results

Table 1 shows the impact on audio recording ratings following file audit reviews. Only ratings that changed as a result of file audit are included. For all other items, the file audit scores were the same as those of the audio recording.

**Table 1:** **Comparison between ratings on the clinical files and audio**-**recordings using the AIMhi Adherence Scale**

Client no.	Items	File audit scores	Audio recording scores
*Client A*	Item 4: Family	9 (Detailed information about the family was noted in the file. The screening phase included discussion of family. This discussion was then revisited briefly prior to treatment)	8 (Item not delivered within 10 mins as suggested in the protocol)
	Item 5: Strengths	8 (Detailed information was noted in the file. The screening phase included discussion of strengths. This discussion was then revisited briefly prior to treatment)	5 (Spoke about strength but did not hear detailed exploration of the strengths of the client)
	Item 6: Stressors/Worries	9 (A pictorial care plan was included which identified stressors, and linked well-being and stressors. The ‘strengths tree’ had circles and lines indicating that a discussion had occurred)	5 (Did not hear the identification of client stressors or elaborations of client’s care plan)
*Client B*	Item 4: Family	5 (Client’s family members and friends were recorded)	Not heard
	Item 5: Strengths	7 (Key strengths and enjoyable activities were recorded)	5 (Spoke about strength but did not hear detailed exploration of the strengths of the client)
	Item 6: Stressors/Worries	9 (A pictorial care plan was included which identified stressors, and linked well-being and stressors)	5 (Spoke about strength but did not hear detailed exploration of the strengths of the client)
	Item 9: Early warning signs	7 (Early warning signs were recorded but there was no plan to deal with these signs of stress)	Not heard
*Client C*	Item 4: Family	9 (Recorded in detail)	5 (Spoke about family but did not hear detailed exploration of family members)
	Item 10: Crisis planning	9 (A detailed crisis plan was developed)	5 (Crisis plan was mentioned but not elaborated)

## Discussion

This study reports on the development of the MCP-AS and reveals its potential suitability as a measure of treatment fidelity. The scale was used to measure both video and audio recording of therapy sessions and findings were strengthened by the additional source of information contributed by the file audits.

These pilot study findings suggest that the tool measures fidelity and that the Indigenous therapists achieved a high standard of fidelity in challenging circumstances. The Gold Standard training video was assessed as having consistent maximum scores using the adherence scale. This finding strengthens confidence in the potential validity of the MCP-AS. It suggests that the scale is measuring what it was originally intended to measure. In addition, the application of the scale to two different sources of information (audio-recording and file notes) with similar rating results, provides further evidence that the scale is measuring fidelity of the therapy.

Ratings were high across the three domains of rapport, motivation and self-management, with especially high ratings for positive feedback and engagement, review of stressors, and goal setting items. The relatively low ratings for Item 2 (information sharing) is likely to be due to the positioning of the therapeutic session after initial baseline outcome measures were completed. The introduction of the therapist to the client will have taken place at commencement of the interview, whereas the recording only commenced once the interview shifted to delivery of therapy. Similarly, the discrepancies between file audit and audio recording ratings for Items 4 and 5 may be explained by those items being covered in the initial introduction to the interview (prior to commencement of therapy). As a result, they were recorded in the file while not heard during the audio recording. The relatively low ratings for Items 9 and 10 (early warning signs discussion and crisis planning) is likely to relate to its position at the end of the therapy session. If time constraints and other commitments interrupted the session, these are the items most likely to remain incomplete. These scores were skewed by their lack of completion within the therapy session of Client B.

In the fields of mental health and service research, adherence scales can be used to aid implementation, dissemination, quality assurance of program/therapy, mentoring/supervision, evaluations of programs and process research [33]. Without documentation and/or measurement of a program’s adherence to an intended model, it is difficult to determine whether unsuccessful outcomes were a failure of the model or the clinician or trainer to implement the model as intended by the creator [34]. Borreli and colleagues [28] stated Adherence Scales are an effective method for researchers and clinicians to identify the strengths and weaknesses of a program. The inclusion of Adherence Scales provides an objective and structured means of capturing feedback about training program development and subsequent trainer/clinician delivery. The results of this pilot study suggest the training delivered to the Indigenous research officers appropriately targeted the key elements of the MCP. When used with larger populations the adherence scale can provide feedback that will allow adaptation of training to therapist needs.

### Study limitations

Circumstances changed the direction of the original clinical trial. Given low numbers of recruited participants it was converted in design to a detailed descriptive study. It is acknowledged the research study we report is thus incomplete and for a number of reasons did not achieve its original aims. On the other hand, the development and pilot testing of the fidelity scale was successfully completed and we thus report a preliminary investigation which delivers lessons from the field.

The small sample size and lack of validation and inter-rater reliability analyses limits the conclusions which can be drawn from these pilot study results. Accordingly, we recommend testing the MCP-AS with a larger population to confirm criterion validity and inter-rater reliability of the scale. On the other hand, the use of audio recording, employment of an independent assessor, inclusion of file audits, and use of two therapists in each session, strengthened the methods and provided multiple sources of information and rich qualitative data for assessment. Comparison with a Gold Standard video provided additional confidence in the validity of the new scale, although a limitation of this comparison was the assessor of the gold standard video was not blind to the status of the video. Whilst the assessor was not the developer of the tool, there was probable bias introduced to this assessment given the developer put this video forward as an example of best practice in the therapy. Video recording the treatment sessions could have provided greater detail about the content of sessions, however this inclusion was considered likely to inhibit engagement. A comparison of therapists and participants’ perception of the achieved treatment quality would have provided valuable information to complement this treatment fidelity research.

## Conclusion

The MCP-AS potentially provides a useful measure of adherence to treatment fidelity, and support for clinicians and researchers to deliver MCP in urban, rural, and remote locations. It can be used to support self-assessment of trainee competence levels and ongoing mentoring of clinicians. One future direction for fidelity research is to recognise the likely benefit of simplifying and translating resource-intensive research tools to clinical, training and supervision settings through adaptation of adherence scales such as the MCP-AS.

## Abbreviations

MCP: Motivational Care Planning
MCP-AS: Motivational Care Planning Adherence Scale
AIMhi: Aboriginal and Torres Strait Islanders Mental health initiative.
